# Partial AV Septal Defect: More Than Just a Hole∗

**DOI:** 10.1016/j.jacadv.2022.100020

**Published:** 2022-03-18

**Authors:** Lorna Swan

**Affiliations:** Scottish Adult Congenital Cardiac Service, Golden Jubilee National NHS Hospital, Clydebank, United Kingdom

**Keywords:** adult congenital heart disease, atrioventricular septal defect, surgical outcomes

**Figure:**
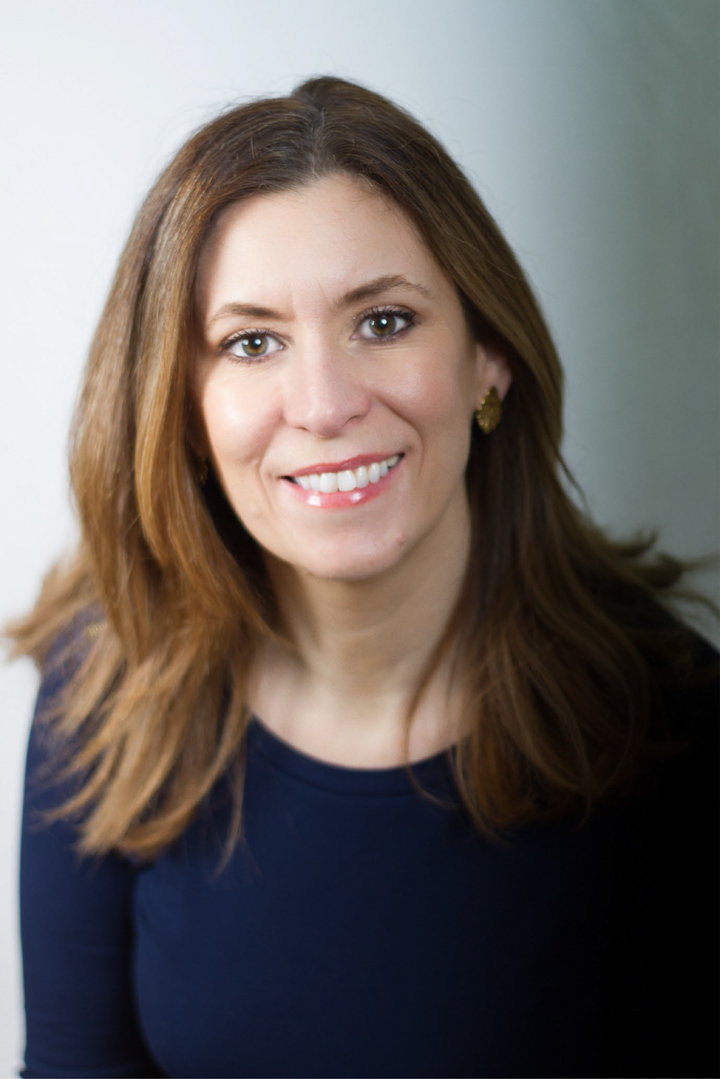

## Not just a hole

From about 33 days in utero, the process of the ventral (anterior) and dorsal (posterior) endocardial cushions fusing starts to occur. Incomplete fusion results in a spectrum of disorders—the atrioventricular (AV) septal defects (also called AV canal defects or endocardial cushion defects). Partial AV septal defects (pAVSDs) occur and then partial fusion occurs, leaving two separate AV valves rather than a common AV junction. Although these patients have two AV valves, these are not mitral and tricuspid valves. The left AV valve usually has 3 leaflets, and the right AV valve may have up to 4 leaflets. Mislabeling the left AV valve as the mitral valve can lead to important misunderstandings of structure and function.

In this issue of *JACC: Advances*, Jain et al^1^ at the Mayo clinic describe their cohort of adult patients with repair of pAVSDs in childhood. All of these patients were born with ostium primum defects, two separate AV valves, and none had large unrestricted ventricular components.

In this follow-up study, the long-term sequelae of operated PAVSDs are clearly seen. This cohort demonstrates a high reintervention rate despite a short median follow-up period of only 4 years. Although this is likely to reflect referral bias to a large tertiary surgical center, it does highlight that PAVSDs are far from a “simple” or hole in the heart. The arrhythmia profile of these patients in adulthood, their risk of reoperation, and their mortality clearly demonstrate that PAVSDs should not be seen as simply a type of atrial septal defect. Reminding ourselves of the embryology of this lesion and focusing on pAVSDs as a defect of the AV junction helps focus our attention on the key components of long-term morbidity: the left AV valve, RV pressure, and the atrial function.

### Early surgical decision-making

At the time of the first operation, 86% of the cohort described in the study by Jain et al^1^ had repair of the left AV valve. Unfortunately, we do not have any information on the nature of this repair or on its subsequent hemodynamic result. We do know that more than half of these patient went on to have reoperation on that valve and one-third ended up with a prosthetic left AV valve despite their young age (median age: 31 years). This has great significance for long-term outcomes including outcomes related to pregnancy.

This cohort was predominantly young women. Decision-making in early life regarding the management of the left AV valve can make future pregnancies complex. Residual left AV valve stenosis or mechanical valves requiring warfarin are especially challenging.

As in other aspects of congenital heart disease, long-term adult outcomes studies, such as this one, form a vitally important continual feedback loop from adult care to pediatric surgical decision-making. In young women, this initial decision-making can be very complex—balancing the detrimental impact of leaving AV valve regurgitation with the risks associated with a post-operatively tight valve or even a valve replacement.

### All arrhythmias are not the same

The atrial arrhythmia burden of this patient group far exceeds that of “more complex” lesions such as tetralogy of Fallot or arterial switch. Although these patients were operated in childhood, by the age of 50 years, 83% of them were having atrial arrhythmias. Atrial arrhythmias were universal over the age of 60 years. This is a distinct picture from that of secundum atrial septal defects where repair at a younger age is associated with a significant reduction in arrhythmia risk when compared to repair over the age of 40 years.^2^

The future arrhythmia profile of these types of patients is important in planning future adult congenital heart disease services as our populations age—investment in ablation and pacing services will become key components of care for these patients—at least until we find truly preventative treatment strategies.

**Figure 1:**
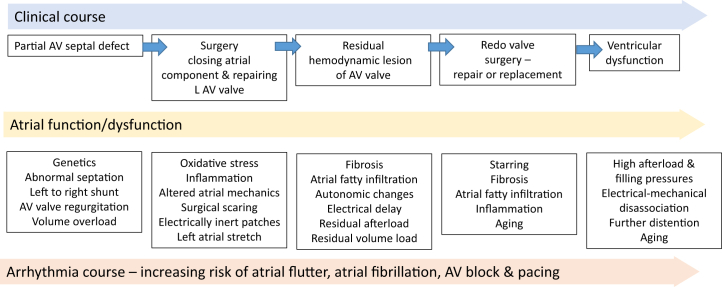
The Inter-Relationship Between Clinical Course, Atrial Function, and Arrhythmia Profile AV = atrioventricular; LAV = left atrioventricular.

This study also demonstrates that all arrhythmias are not created equally. In follow-up studies, it is tempting to lump together all forms of atrial arrhythmia. This study clearly shows why this is inappropriate with atrial fibrillation having a very different significance than atrial flutter. Atrial fibrillation in this setting can be seen as a red flag indicating the increased risks of death, arrhythmia hospitalization, and heart failure. While we do not as yet understand the causality of these associations, the postulated theory of left atrial myopathy (or left atrial “distress”) requires further investigation (Figure 1).

## Conclusions

Many congenital heart lesions are known to be conditions with an almost obligatory need for reintervention—for example the patient with tetralogy of Fallot repaired with a transannular patch. We should approach the patient with PAVSDs with the same mind set—that future intervention is highly probably and that lifelong specialist care in a congenital heart disease service is essential.

## Funding Support And Author Disclosures

The author has reported that they have no relationships relevant to the contents of this paper to disclose.
